# Supplementary material for: A set of multi-entry identification keys to African frugivorous flies (Diptera, Tephritidae)
Source: Zookeys. 2014 Jul 24;(428):97–108. doi: 10.3897/zookeys.428.7366 (PMC4143993; doi:10.3897/zookeys.428.7366)
Supplement: Supplementary material 6 — Key to Ceratitis [file zookeys-428-097-s006.zip › SF6_ZooKeys_key to Ceratitis/key/SF6_ZooKeys_key to Ceratitis/Media/Html/Ceratitis simi .htm]

Ceratitis (Ceratalaspis) simi Munro


***Ceratitis*** **(*Ceratalaspis*)
*simi* Munro**

*Ceratitis* (*Pardalaspis*) *simi*
Munro, 1933a: 37.

Body
length: 6.38 (5.40-7.15) mm; wing length: 6.24 (5.85-7.25) mm.

Male

Head.
Antenna yellow to yellow-orange. Third antennal segment twice as long as second
segment. Arista with very short hairs over entire length. Frons with short
scattered hairs which are distinctly darker than the frons; concave to flat, in
lateral view slightly projecting forwards at antennal implant. Face usually
yellow-white, but sometimes somewhat darker, more yellow. Darker patches at
lower eye margin, but not always outspoken. Chaetotaxy normal for subgenus.

Thorax.
Postpronotum white or white-yellow, spotted. Ground colour of mesonotum pale
with orange tinge. Chaetotaxy normal for subgenus. Scapular setae black. One or
two anepisternal bristles. Scutellum white basally, otherwise yellow with three
black separate markings apically; basally two dark but inconspicuous spots.
Subscutellum pale with three dark spots, touching along dorsal margin.

Legs
yellow; setation typical for subgenus, mainly pale especially on femora.
Posterior and posterodorsal rows on fore femur pale.

Wing
bands with markings extensively yellow. Banding, marginal band continuous;
cubital band joined with discal band; medial band free; crossvein r-m at middle
of discal cell. Crossvein dm-cu position variable.

Abdomen.
Dark yellow colour. Setation and banding typical for subgenus.

Female

As
male except for the following characters: Scutellum with basal spots more
outspoken. Oviscape shorter than abdominal terga 3-6 combined.

(Description
after De Meyer, 1998)
